# Twenty-eighth Annual Meeting of the Illinois State Medical Society

**Published:** 1878-06

**Authors:** N. S. Davis


### Society Reports.

---

### THE TWENTY-EIGHTH ANNUAL MEETING OF THE ILLINOIS STATE MEDICAL SOCIETY, SPRINGFIELD, MAY 21ST AND 22ND, 1878.

*(Reported for The Chicago Medical Journal and Examiner by N. S. Davis, M. D.)*

The delegates and members of the Illinois State Medical Society assembled in regular annual session on the 21st day of May, 1878, in the Representatives' Hall at the Capitol in Springfield, and were called to order at 10 o'clock a. m., by Dr. J. L. White, of Bloomington, President of the Society. Prayer was offered by the Rev. Mr. Fullerton, and an address of welcome in behalf of the Committee of Arrangements, was delivered by Dr. B. M. Griffith, Chairmain of the Committee. The list of Standing and Special Committees was called, and the reports and papers assigned to particular hours for reading. Dr. J. F. Todd called the attention of the Society to the death of the late G. W. Crossley, of Princeton; and on motion of Dr. T. D. Fitch, the President was requested to appoint a committee of three to report resolutions of respect for the memory of Dr. Crossley. The President appointed Drs. J. F. Todd, T. D. Fitch and T. F. Worrell such committee.

The President, Dr. J. L. White, of Bloomington, delivered his annual address, which was listened to with pleasure and profit, and a copy requested for publication in the transactions of the Society. Governor Cullom having come into the hall to listen to the address of the president, was at its close escorted to the platform and introduced to the Society by the vice-president, Dr. E. P. Cook, of Mendota. The Governor responded in a brief and very appropriate address, in which he alluded to the establishment of a State Board of Health, and the operation of the law to regulate the practice of medicine in this State. He stated that the doubts entertained at first, in regard to the propriety of those measures, had been entirely removed by their practical operation thus far, and he should give them a cordial support. His address was listened to with much pleasure. Dr. T. D. Washburn, of Hillsborough, then read a brief paper highly eulogizing the medical laws establishing the State Board of Health and regulating the practice of medicine, which was received and referred to the Committee of Publication.

### Afternoon Session.

The Society was called to order by the president at 2:30 p. m.

The Censors reported on several candidates who had been proposed for election as permanent members, and they were unanimously elected.

Dr. T. D. Fitch, chairman of the committee on the revision of the constitution and by-laws, made a report embodying a complete revised constitution and by-laws, which were read, section by section, and adopted with but little discussion. The chief items of difference between it and the old constitution, are, the omission of the provision for selecting *permanent members;* the insertion of a clause providing for honorary membership by non-residents of the State ; and another providing for a permanent *Judicial Council*, in the place of the Board of Censors. The constitution and by-laws as adopted were referred to the Committee of Publication, with instructions to publish them in the Transactions, and also a separate edition in pamphlet form.

Dr. D. Prince, of Jacksonville, presented and explained the operation of a galvanometer, for measuring or indicating the intensity of the galvanic current during the application of galvanism or electricity to medical and surgical purposes.

Dr. N. Wright, of Chatham, read a report on the action of malaria and the treatment of its effects ; the chief peculiarity of which was the advocacy of hypodermic injections of sulphate of morphia, to suppress or mitigate the paroxysms of an intermittent fever. He claimed that the hypodermic injection of a moderate dose of morphine at the commencement of the cold stage always cut that stage short, and rendered the hot and sweating stages very slight. He did not claim that it prevented the recurrence of the paroxysms. The paper led to an interesting discussion concerning the best mode of treating chronic agues and other effects of malaria, during which Dr. N. S. Davis advocated extreme caution in the use of morphine and other active narcotics hypodermically.

Dr. T. D. Fitch, of Chicago, Chairman of the Committee on Obstetrics and Diseases of Women, read an interesting report, after which the society adjourned to 9 o'clock Wednesday morning.

### Wednesday Morning Session.

The Society was called to order at 9 o'clock A. M., by the President.

The report of the Committee on Obstetrics being in order, Dr. E. R. Willard, of Wilmington, read a paper on puerperal eclampsia, and Dr. Ellen Ingersol, of Canton, read an additional report on some of the more common matters, that are apt to be neglected in the management of ordinary labors. On motion of Dr. F. B. Watts, the report of the Committee on Obstetrics, and the accompanying papers, were received and referred to the Committee of Publication.

A recess of ten minutes was taken, to allow the members from each county to select one of their number as a member of the Committee on Nominations; after which Dr. C. W. Earle, of Chicago, read the report of the Standing Committee on Practical Medicine. It was followed by a discussion participated in by Drs. S. O. Richey, J. S. Jewell, S. J. Jones, E. L. Holmes, of Chicago; N. B. Buck, of Springfield; and Dr. Johnson, of Peoria. The report was referred to the Committee of Publication.

Dr. Moses Gunn, of Chicago, Chairman of the Standing Committee on Surgery, read an interesting report, which was received and discussed by Drs. E. Andrews, E. Ingals, N. S. Davis, of Chicago; D. Prince, of Jacksonville; and H. C. Gill, of Jerseyville, and referred to the Committee of Publication.

Dr. N. S. Davis, chairman of the Committee on Drugs and Medicines, read a brief report, which, after some remarks by Dr. J. H. Hollister, was referred to the Committee of Publication.

Dr. J. F. Todd, on behalf of the special committee on the death of Dr. G. W. Crossley, reported the following preamble and resolutions, which were adopted and ordered published:

WHEREAS, We recognize in the death of our professional brother, Dr. G. W. Crossley, a serious loss to our society and the medical profession, as a tribute of respect for his manly character,

*Resolved*, That while we bow in humble submission to the afflictive dispensation, we are comforted with the knowledge that he passed through the last great ordeal with the firmness and composure of a noble nature, sustained by exalted Christian sentiments.

*Resolved*, That we cherish his memory for the uniform and gentle courtesies he constantly bestowed upon us during life, and for his unselfish and intelligent devotion to his profession.

*Resolved*, That his unsullied reputation, and the purity of his life, be ever held in our estimation as an example worthy of our respect and emulation.

*Resolved*, That we extend our warmest sympathy to his family and friends in their great bereavement.

*Resolved*, That a copy of these resolutions be sent to the family of the deceased, and offered for publication in the Princeton papers.

Dr. T. F. Worrell, Chairman of the Committee on Nominations, reported, recommending the election of the following officers and committees for the coming year:

For *President*, Dr. E. P. Cook, of Mendota.

*Vice-Presidents*, Drs. James S. Whitmire, of Metamora, Geo. W. Jones, of Danville.

For *Treasurer*, Dr. J. H. Hollister, of Chicago.

*Assistant Secretary*, Dr. R. P. Wilson, of Lincoln.

*Committee of Arrangements:* Drs. R. P. Wilson, C. T. Wilbur, L. L. Leeds, P. L. Dieffenbacker, N. S. Reed.

*Judicial Council:* Drs. F. B. Haller, of Vandalia; E. Ingals, P. H. Burton, Rob't Boal, E. R. Willard, A. T. Darrah, T. D. Fitch, C. Goodbrake, N. S. Read.

*Committee on Practical Medicine:* Drs. G. W. Jones, of Danville; J. S. Crow, L. B. Moore.

*Committee on Surgery:* Drs. John E. Owens, of Chicago; M. M. Deming, H. Z. Gill.

*Committee on Obstetrics:* Drs. C. C. Hunt, of Dixon; D. S. Booth, W. M. Kaull.

*Committee on Gynecology:* Drs. T. D. Fitch, of Chicago; P. L. Dieffenbacker, N. Budge.

*Committee on Ophthalmology and Otology:* Drs. S. J. Jones, of Chicago: J. Perrin Johnson, H. B. Young.

*Committee on Drugs and Medicines:* Drs. J. F. Todd, C. B. Johnson, John Wright.

*Committee on Necrology:* Drs. T. F. Worrell, of Bloomington; G. W. Albin, E. Ingals.

*Committee on Syphilis:* Dr. W. M. Chambers, of Charleston.

*Committee on Psychological Medicine:* Dr. A. McFarland, of Jacksonville.

*Committee on Physical Science:* Dr. Sarah H. Stevenson.

*Committee on Croup:* Dr. H. Y. Gill.

*Committee on Medical Education:* Drs. E. Ingals, of Chicago; R. G. Bogue, of Chicago; D. Prince, of Jacksonville.

Dr. C. C. Hunt, of Dixon, Chairman of the Committee on Diseases of Children, read a report, which was supplemented by a paper on cholera infantum, by Dr. Lucinda H. Carr, a member of the same committee. The report and accompanying paper were referred to the Committee of Publication.

Dr. J. S. Jewell, a Special Committee on Neurology, made a verbal report on the nature and treatment of epilepsy. On motion of Dr. W. M. Chambers, he was requested to furnish a written copy of his address for publication in the Transactions. The remainder of the afternoon session was occupied with the hearing of a lengthy report on croup, by Dr. H. Y. Gill, of Jerseyville.

### Evening Session.

The Society was called to order at 8 o'clock p. m., by the President. The consideration of the report on croup being in order, it was referred to the author with a request that he prepare an abstract for the Transactions of the Society, and that he be authorized to publish the report in full, in a separate volume as his own property.

Dr. C. W. Earle presented the report on Electro-Therapeutics by Dr. P. S. Hayes, of Chicago, and it was referred to the Committee on Publications. Short papers were also read by Dr. S. J. Jones, of Chicago, on Otology, and on Diseases of the Lachrymal Passages; by Dr. E. L. Holmes of Chicago, on Ophthalmology; by Dr. E. Andrews of Chicago on an Instrument for Detecting Fragments of Stone in the Bladder by Auscultation; by Dr. S. O. Richey of Chicago, on Reparation of Imperfect Tympani; by Dr. Mathews of Carlinville, on Diseases of the Throat and Nasal Passages; and by Dr. W. T. Montgomery of Chicago, on Two Cases of Disease of the Eye; all of which were briefly discussed and referred to the Committee of Publication.

At the request of Dr. D. W. Graham, of Chicago, a committee was appointed to assist the publishers of the *Medical Register and Directory* of the State, consisting of Drs. E. P. Cook, of Mendota, F. B. Huller, of Vandalia, and Wm. M. Chambers of Charleston.

A communication from the Centennial Medical Society was presented, relating to physicians' bills and to fees for services as expert witnesses in courts of justice. It was referred to a special committee of three, consisting of Drs. Eli Bowyer of Olney, T. F. Worrell of Bloomington, and H. Y. Gill of Jerseyville; to report at the next annual meeting of the society.

Lincoln, Logan county, was selected as the next place of meeting. A full list of delegates was appointed to the meeting of the American Medical Association for 1879. Visiting delegates were also appointed to the State Medical Societies of Michigan, Indiana, Missouri, Iowa, and Kentucky. At a late hour in the evening the Society adjourned *sine die.*

All the sessions were well attended, delegates and members being present from more than forty counties in the State. The business throughout was transacted in good order, and the meeting will be remembered as one of the most pleasant and profitable in the history of the Society.

